# Functional Benefit and Orthotic Effect of Dorsiflexion-FES in Children with Hemiplegic Cerebral Palsy

**DOI:** 10.3390/children10030531

**Published:** 2023-03-09

**Authors:** Idan Segal, Sam Khamis, Liora Sagie, Jacob Genizi, David Azriel, Sharona Katzenelenbogen, Aviva Fattal-Valevski

**Affiliations:** 1 Pediatric Neurology Institute, Dana-Dwek Children’s Hospital, Tel Aviv Sourasky Medical Center, Tel Aviv 6093246, Israel; 2 Pediatric Neurology Unit, Emek Medical Center, Afula 1834111, Israel; 3 The Gait and Motion Analysis Laboratory, Department of Pediatric Orthopaedics, Dana-Dwek Children’s Hospital, Tel Aviv Sourasky Medical Center, Tel Aviv 6093246, Israel; 4 Pediatric Neurology Unit, Bnei-Zion Medical Center, Haifa 3339419, Israel; 5 Rappaport Family Faculty of Medicine, Technion—Israel Institute of Technology, Haifa 3200003, Israel; 6 Faculty of Industrial Engineering and Management, Technion—Israel Institute of Technology, Haifa 3200003, Israel; 7 Sackler Faculty of Medicine, Tel Aviv University, Tel Aviv 6997801, Israel

**Keywords:** hemiplegia, cerebral palsy, FES, functional benefit

## Abstract

Functional electrical stimulation of the ankle dorsiflexor (DF-FES) may have advantages over ankle foot orthoses (AFOs) in managing pediatric cerebral palsy (CP). This study assessed the functional benefit and orthotic effect of DF-FES in children with hemiplegic CP. We conducted an open-label prospective study on children with hemiplegic CP ≥ 6 years who used DF-FES for five months. The functional benefit was assessed by repeated motor function tests and the measurement of ankle biomechanical parameters. Kinematic and spatiotemporal parameters were assessed by gait analysis after one and five months. The orthotic effect was defined by dorsiflexion ≥ 0° with DF-FES at either the mid or terminal swing. Among 26 eligible patients, 15 (median age 8.2 years, range 6–15.6) completed the study. After five months of DF-FES use, the results on the Community Balance and Mobility Scale improved, and the distance in the Six-Minute Walk Test decreased (six-point median difference, 95% CI (1.89, 8.1), –30 m, 95% CI (−83.67, −2.6), respectively, *p <* 0.05) compared to baseline. No significant changes were seen in biomechanical and kinematic parameters. Twelve patients (80%) who showed an orthotic effect at the final gait analysis experienced more supported walking over time, with a trend toward slower walking. We conclude that the continuous use of DF–FES increases postural control and may cause slower but more controlled gait.

## 1. Introduction

Cerebral palsy (CP) defines a group of permanent disorders of movement and posture that are attributed to non-progressive disturbances in the developing brain [1]. Hemiplegic CP accounts for 21–40% of all cases of CP [2,3]. Children with hemiplegic CP are typically ambulant with high motor functioning (Gross Motor Function Classification Scale (GMFCS) I/II), but with asymmetry of gait and a greater risk of instability and falling [4]. The ankle joint is affected in virtually all patients, causing insufficient clearance of the foot during the swing phase (“foot drop”) and abnormal foot contact during the stance phase of gait [5].

Children with hemiplegia show deviations in their spatiotemporal gait parameters. Their gait is asymmetric, as manifested by a shorter stance phase on the affected side compared to the unaffected side. In addition, these children have slower walking speeds and a more supported gait, with a longer double support phase (when both feet are in contact with the ground) than typically developing children. [6,7].

Ankle–foot orthoses (AFOs) are usually prescribed to improve foot positioning and prevent foot drop in these children [8]. However, while AFOs have known benefits, compliance deteriorates as children get older [9].

Functional electrical stimulation (FES) is a well-known neuroprosthesis that delivers electrical stimulation to the motor nerve and activates the desired muscle group. Dorsiflexion FES (DF-FES) stimulates the common peroneal nerve and activates the ankle dorsiflexors, correcting upper motor neuron foot drop [10]. Theoretically, the repetitive stimulation of the DF muscle may give DF-FES an advantage over AFOs, which passively support the ankle and may increase muscle atrophy [8,11,12]. Indeed, studies have shown improvement in ankle kinematic parameters with DF-FES (this is typically referred to as an orthotic effect). However, the orthotic effects of DF-FES may vary between children [13,14]. There is no consensus on what clinical features can predict appropriate candidates for DF-FES, although an adequate ankle range of motion (ROM) was suggested as a prerequisite [15].

In addition, studies in adults and children have pointed to a “therapeutic effect” after the continuous use of DF-FES, meaning improvement in any aspect of gait, including biomechanical or other functional parameters, which continues when the patient is not using the DF-FES device. Some studies have shown peripheral improvement in ankle biomechanical parameters, e.g., ankle ROM, muscle strength and size, and spasticity [6,7,8,9,10,11,12,13,14,15,16,17,18]. Studies have also shown improved balance scores [8,18,19,20,21,22]. However, studies examining a kinematic therapeutic effect have reached conflicting conclusions [11,18,19]. In addition, the findings on the effects of continuous DF-FES use on spatiotemporal parameters and walking speed are scarce and mixed [19,21,23]. Finally, the long-term therapeutic effect of DF-FES—namely, whether it has a prolonged carry-over effect (without the device) or whether improvements are only temporary—remains a major open question [15].

The aim of this study was to evaluate the functional and therapeutic effects of DF-FES use over five months in children with hemiplegic CP, including effects on postural control; walking endurance and speed; and ankle biomechanical, spatiotemporal, and kinematic parameters. In the rest of this paper, the effects observed with DF-FES turned on will be called functional effects or benefits, while the effects observed with DF-FES turned off will be called therapeutic effects.

In addition, we looked for clinical, kinematic, and biomechanical parameters that may serve as predictor(s) for the achievement of an orthotic effect. Previous studies [13,18,19,24,25,26] used improvement in any ankle swing kinematic parameters (e.g., peak dorsiflexion angle) to indicate an orthotic effect. We introduce a more precise measure for the orthotic effect of DF-FES. In this study, an orthotic effect was defined by whether it prevents excessive swing plantarflexion (>1 SD from the mean)—i.e., foot drop—and achieves swing dorsiflexion ≥0° at the mid or terminal swing phase.

## 2. Materials and Methods

### 2.1. Study Design

This was a prospective open-label study on hemiplegic CP children with foot drop who used DF-FES (WalkAide; Innovative Neurotronics, Austin, TX, USA) for five months. Motor function tests were conducted at baseline, after one month, and after five months of device use. Motor tests included the Community Balance and Mobility Scale (CB&M), the Six-Minute Walk Test (6MWT), and the Timed Up and Down Stairs Test (TUDS) (see details below).

Falling questionaries were filled in by parents at baseline and at the end of the study. In addition, at the end of the study, children filled in satisfaction questionaries. Biomechanical ankle parameters (see below) were taken at baseline and at the end of the study.

**Figure 1:**
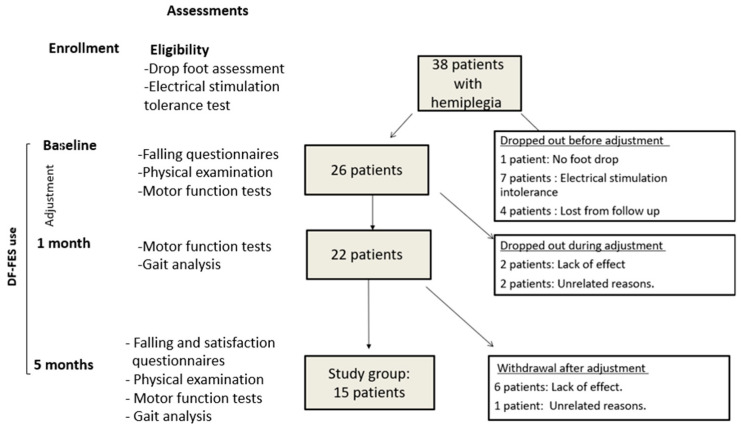
Flow chart and study design.

To test for the presence of an orthotic effect, we conducted gait analysis with DF-FES switched off and switched on. On the assumption that the device requires adjustment, in order to assess the true orthotic effect, the first gait analysis was conducted after one month. Gait analysis was repeated after five months of device use, allowing us to assess changes in kinematic and spatiotemporal parameters (Figure 1).

To assess predictors for failure or success in the achievement of an orthotic effect (namely, swing dorsiflexion ≥0° at the mid or terminal swing phase), the clinical kinematic and biomechanical parameters of children who showed vs. did not show an orthotic effect were compared (see below).

### 2.2. Study Population

The inclusion criteria comprised children (≥6 years) with hemiplegic CP, GMFCS I/II, and foot drop. The exclusion criteria were fixed ankle joint contracture (passive ROM < 0° with knee extended); an inability to tolerate the electrical stimulation of DF-FES; orthopedic surgery or botulinum toxin injection to the lower limbs within six months of enrollment; moderate to severe intellectual disabilities; or uncontrolled epilepsy. For the power calculation, we used Shieh’s [27] method. For a power of 0.8 with alpha = 0.05, based on previous studies [18,19], and assuming a median-difference-to-standard-deviation ratio of 0.8 in motor function tests, the required sample size was calculated as between 11 (Laplace distribution) and 14 (uniform distribution). The final sample in our study comprised 15 patients (see Results Section 3 and Figure 1).

### 2.3. Enrollment

The passive ankle range of motion (to ensure passive ROM ≥ 0° with knee extended), tolerance of DF-FES, and foot drop were assessed at enrollment to determine inclusion (Figure 1). Foot drop was defined by excessive swing plantarflexion during the mid or terminal swing (>1 standard deviation (SD) from the mean). During enrollment, patients were asked to walk barefoot for 15 m using the RehaGait mobile gait analysis system (RehaGait, HASOMED GmbH Service, Magdeburg, Germany). This is a mobile clinical tool equipped with motion sensors which utilize the inertia of the mass to detect movement changes. The sensors include a three-axis accelerometer for recording linear acceleration, a gyroscope for recording angular velocity, and a magnetometer for recording orientation in relation to the earth’s magnetic field; the system measures kinematics and angles while producing a graphical presentation of the gait cycle. In previous studies [28,29], measures of sagittal plane joint kinematics produced by this system were comparable to those from a camera-based system. A graphical presentation of the gait cycle (with upper and lower borders +/−1 SD) was used to assess excessive plantarflexion (>1 SD) during the mid/terminal swing.

### 2.4. Motor Function Tests

Motor function tests were conducted at baseline, after one month, and after five months. All tests were carried out by the same physiotherapist, in the same order, for all patients, and at each visit. Each test was conducted with the device attached to the patient’s leg. At baseline (before adjustment), tests were carried out with the device switched off, and at one and five months, each test was carried out first with the device switched off and then with it switched on. Motor tests included the Community Balance and Mobility Scale (CB&M) [30], the Six-Minute Walk Test (6MWT) [31], and the Timed Up and Down Stairs Test (TUDS) [32].

#### 2.4.1. Community Balance and Mobility Scale (CB&M)

The CB&M is a clinical tool used to assess postural stability and dynamic balance. It includes tasks that are representative of the motor skills thought to be necessary for everyday functioning in community settings. It has been used in adults and children with acquired brain injury and also in high-functioning children with hemiplegic CP who reach a ceiling effect on other objective measures such as the Gross Motor Function Measure (GMFM). The CB&M comprises a total of 13 tasks, with 6 items measured bilaterally. Each task is rated on a six-point scale (0–5), with one item allowing for a bonus point. The highest possible score is 96. A change of five points is considered clinically meaningful [18].

#### 2.4.2. Timed up and down Stairs Test (TUDS)

The TUDS is a functional mobility test used to assess postural control [32]. The patients were asked to walk up and then down an 11-step flight of stairs. The steps were 18.5 cm high and 31 cm deep; no stickers or other means were used to help patients determine the depth or height. At the start of the test, the patients were asked to stand 30 cm from the bottom step. They were then instructed to go up quickly but safely, to turn around on the top step, and to descend until both feet were on the bottom step. The patients were allowed to choose any method of traversing the stairs, including using a handrail. The TUDS score was the time in seconds from the start cue until the second foot returned to the bottom step [30]. To the best of our knowledge, no minimal meaningful change for this test has been published for the cerebral palsy population.

#### 2.4.3. Six-Minute Walk Test (6MWT)

The 6MWT has been reported to reflect functional capacity in terms of activities of daily living in the cerebral palsy population [31]. The patients were instructed to walk as far as possible in a straight line for six minutes along a 10 m course, without running or jogging. They were permitted to slow down or stop to rest but were instructed to resume walking as soon as they could. Masking tape was placed at two-meter intervals along the course, and the distance covered in six minutes was recorded to the nearest meter [31]. Thompson et al. [33] reported minimal meaningful changes for school-aged children with cerebral palsy of 61.9 m and 64.0 m for GMFCS Levels I and II, respectively.

Overall, we hypothesized that patients using DF-FES continuously would show a functional benefit manifested by an improvement in stability and balance, a greater distance in the 6MWT due to increased walking endurance and/or speed, and faster speed walking up and down stairs.

### 2.5. Falling and Satisfaction Questionnaires

The parents filled out a questionnaire in which they scored their child’s falling frequency as daily, weekly, monthly, or less at baseline and at the end of the study. At the end of the study, the children were asked to rate their satisfaction with the device on a scale of 1–5 (1 = very much, 5 = not at all).

### 2.6. Ankle Biomechanical Assessments

At baseline and after five months of device use, four assessments—plantarflexor muscle spasticity, dorsiflexor muscle strength, muscle selectivity, and a precise measurement of passive ankle ROM (which was tested at enrollment only for exclusion criteria)—were carried out by a physiotherapist, as follows:

1. Passive ankle range of motion with knee flexion and extension (with the leg supported on a bed), with the subtalar joint maintained in a neutral position. The measurement was conducted using a goniometer aligned at one end with the fibula and at the other end with the fifth metatarsal bone [34]. Foot deformities such as midfoot break were accounted for by accurately measuring calcaneal dorsiflexion with the foot held in supination.
2. Plantar-flexor muscle spasticity with knee flexion and extension (for soleus and gastrocnemius muscle assessment, respectively). The measurement was conducted by dorsiflexion of the foot from maximum possible plantarflexion to maximum possible dorsiflexion. Spasticity was scored using the modified Ashworth scale [35].
3. Muscle selectivity. Ankle joint selectivity was measured using the Selective Control Assessment of the Lower Extremity (SCALE), with patients in a sitting position with the knee extended. Patients were asked to move their foot up, down, and up again. Selectivity was scored based on a three-point scale (zero points = unable, one point = impaired, two points = normal) [36].
4. Dorsiflexor muscle strength. Strength was evaluated in side-lying and seated positions using Kendall’s manual muscle testing scale [37].

### 2.7. Dorsiflexion-Functional Electrical Stimulation Device (DF-FES)

The WalkAide device (WalkAide; Innovative Neurotronics, Austin, TX, USA) is a small device which is attached to the patient’s leg by a cuff that sits just under the knee on the affected side. One electrode is placed on the belly of the tibialis anterior muscle, and the other on the common peroneal nerve. Electrical stimulation is triggered by a tilt sensor which senses the change in the tibia angle during the swing phase [24].

For each patient, at baseline setup, the pulse width was adjusted to between 25 and 50 microseconds, and the frequency range was adjusted to between 16.7 and 33 pulses/s, in order to achieve ankle dorsiflexion with tolerable stimulus. During the one-month adjustment period, families were instructed to (a) gradually increase the intensity of electrical stimulation according to the child’s tolerance in order to maximize ankle dorsiflexion; and (b)to reach minimal requirements for constant DF-FES use of at least, on average, 5 days/week, 4 h/day and 1500 steps/day (75% of the average steps per day in our preliminary tests). Compliance with these requirements was confirmed via the device’s internal log, which recorded stimulations (“stims”) and hours of “device on” per day.

The patients were instructed to maintain good hygiene in order to avoid skin irritation and burns beneath the electrodes.

### 2.8. Gait Analysis

Gait analysis was conducted at the end of the first month of device use (allowing for adjustment) and was repeated at the end of the study after five months of use. Gait analysis was performed in the laboratory using a three-dimensional motion analysis system (the Plug-in Gait model (PGM) by Vicon^®^, Oxford Metrics, UK) with a sampling rate of 120 Hz. The Plug-in model provides full upper or lower body joint kinematics and kinetics modeling using a pre-defined Plug-in Gait marker set. These retro-reflective markers were applied to anatomical landmarks to capture gait performance [38]. Where a midfoot break was identified, the forefoot marker was placed proximally along the foot axis toward the hindfoot in order to avoid measuring midfoot dorsiflexion (as opposed to ankle dorsiflexion).

The children were asked to walk barefoot at a self-selected speed along a 14 m walkway. Ten representative gait cycles were captured from five gait trials conducted with FES turned off, and another ten cycles were captured from five trials conducted with FES turned on. Ankle kinematic parameters were analyzed at defined points in each cycle: initial contact angle; maximal and minimal dorsiflexion angle at the mid and terminal swing; and maximal ankle dorsiflexion at mid-stance as an indication of ankle ROM during the weight-bearing state [39]. Video recordings of the trials were used to evaluate the heel strike. We hypothesized that DF-FES would improve kinematic parameters and correct swing foot drop (i.e., produce an orthotic effect) in those with a better ankle ROM.

In addition, spatiotemporal parameters were analyzed, including stance time (in sec) and percentage of gait cycle; walking speed; cadence (steps/min); step time (in sec) and length (in cm); and single and double support time (in sec). Although data in the literature are scarce, we hypothesized that if DF-FES has a beneficial effect on the spatiotemporal parameters of gait, we should expect a trend toward the normalization of gait deviations when comparing the final gait analysis to the first one, including reduced supported gait (with a decrease in the double/single support time ratio), an increased walking speed, and an increase in the percentage of the stance of the gait cycle.

### 2.9. Orthotic Effect

In this study, the orthotic effect was defined by whether it prevents excessive swing plantarflexion (>1 SD from the mean)—i.e., foot drop—and achieves swing dorsiflexion ≥0° at the mid or terminal swing phase. To test for an effect, we compared the minimal and maximal dorsiflexion angles at the mid and terminal swing using the medians of the 10 gait cycles captured with DF-FES turned off and on. An orthotic effect was considered present (OE+) when DF-FES produced dorsiflexion (≥0°) at either the mid or terminal swing and absent (OE–) when no such change appeared.

### 2.10. Statistical Analyses

The results of repeated motor function tests, the kinematic and spatiotemporal parameters, and the ankle biomechanical parameters of the 15 patients who completed the study protocol were compared by the Wilcoxon signed-rank test. CB&M scores were compared only for 14 patients because of low cooperation by one girl (6.5 years old). In order to define the right candidates for DF-FES, demographic, clinical (e.g., AFO use and history of botulinum toxin injection), and physical parameters (e.g., ankle spasticity using the Modified Ashworth Scale (MAS) and ROM) were compared between the study group and patients who dropped out. In addition, demographic, clinical, physical, kinematic, and device-use parameters were compared between patients who did and did not experience an orthotic effect according to the first gait analysis, using Fisher’s exact test for categorical variables (e.g., age, GMFCS, MAS score, muscle strength, and selectivity) and the Mann–Whitney test for continuous variables (e.g., ankle ROM, ankle kinematic parameters, and device use parameters (stims and hours/day)) (see Supplementary Table S2). Since most patients who did not experience OE dropped out during the study, this analysis could not be conducted at the final gait analysis. The diagnostic value of various parameters as predictors for an orthotic effect was assessed using the Pearson correlation coefficient. MedCalc Statistical Software version 20.115 (MedCalc Software, Ostend, Belgium; https://www.medcalc.org (accessed on 1 January 2023); 2020) was used for the statistical analyses.

## 3. Results

### 3.1. Clinical and Demographic Variables

Among 38 patients with hemiplegia who were tested for eligibility, 26 patients met the inclusion criteria and began to use DF-FES according to the adjustment instructions. Twenty-two patients completed the first month of use according to the minimal requirements and underwent gait analysis. Fifteen patients (the study group) completed five months of device use and repeated the motor function tests, biomechanical tests, and gait analyses. Those 15 patients showed good compliance, with an overall average of 3802 ± 790.8 stims/day, 21.6 ± 4.86 days/month, and 6.72 ± 1.55 h/day. Satisfaction questionnaires showed a high satisfaction (average 1.82 ± 1.25 on a scale from 1 (very much) to 5 (not at all)).

Of the 11 patients who dropped out between enrollment and the five-month mark, eight patients (72%) withdrew due to a lack of a positive effect of FES on gait (as perceived by the patient and family), and three (28%) withdrew for unrelated reasons (Figure 1). Comparing the clinical and demographic variables, a history of botulinum toxin injections was more prevalent in patients who dropped out (*n* = 11) than in the study group (73% vs. 20%, respectively; *p* = 0.01) (Table 1).

### 3.2. Falling

According to the questionnaires filled out by the parents, 12 patients in the study group (60%) fell frequently (daily or weekly) at baseline vs. 3 (20%) after five months of FES use (*p* = 0.06).

### 3.3. Motor Function Tests

#### 3.3.1. Community Balance and Mobility Scale (CB&M)

**Figure 2:**
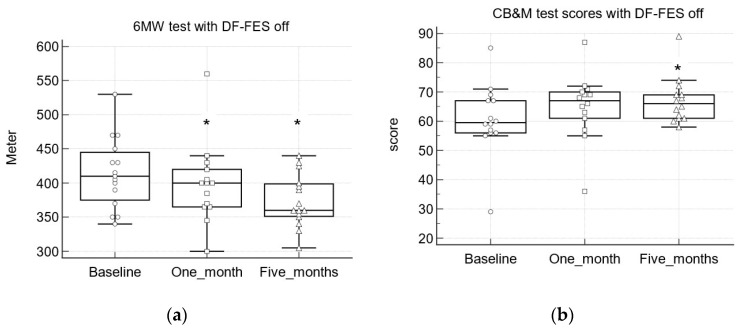
Repeated motor function test (**a**) and scores (**b**) test scores with DF-FES off, at baseline and after one month and five months of device use. Error bars represent 95% CI of median. Dots represent individual data. CB&M = Community Balance and Mobility Scale; 6MWT = Six-Minute Walk Test; * *p* < 0.05 in comparison to baseline (Wilcoxon test).

The CB&M measures postural stability and dynamic balance. Repeated CB&M tests at baseline, after one month, and after five months of FES use demonstrated improvement, with higher scores that reached statistical significance after both one month and five months. Comparing the baseline (DF-FES off) to the final test after five months (device on and off), we found median differences of 6.5 (95% CI (2.79, 10), *p <* 0.01) and 6 (95% CI (1.89, 8.1), *p <* 0.01) with DF-FES switched on and off, respectively. Between one and five months, there was a trend toward improvement, with increased median differences, although these findings were not significant (Table 2; Figure 2).

#### 3.3.2. Timed up and down Stairs Test (TUDS)

The TUDS assesses postural control, and improvement should be manifested by faster speeds in climbing up and down stairs. Our findings showed only a minor trend in this direction. The median differences in seconds between five months (FES on and off) and baseline (FES off) were −0.41 (95% CI (−0.27, 0.28), *p* = 0.06) and −0.83 (95% CI (−2.28, 0.42), *p* = 0.09) for FES on and off, respectively (Table 2).

#### 3.3.3. Six-Minute Walk Test (6MWT)

The 6MWT is used to measure functional ability. According to previous results [17] and our hypothesis, improvement should be manifested by an increase in walking distance. However, our study showed the opposite trend. Repeated 6MW tests at baseline, after one month, and after five months of FES use revealed a decrease in walking distance over time, which reached statistical significance after one month with DF-FES switched on and after five months with the device both on and off (Table 2, Figure 2). The median differences in distance between five months (DF-FES on and off) and baseline (DF-FES off) were −35 m (95% CI (−99.67, −3.97), *p <* 0.05) and −30 m (95% CI (−83.67, −2.6), *p <* 0.05) with DF-FES on and off, respectively. The trend toward reduced distance continued between one and five months but did not reach statistical significance (Table 2).

### 3.4. Kinematic, Spatiotemporal, and Biomechanical Parameters

#### 3.4.1. Orthotic Effect

The study sample (N = 15) exhibited significant improvement in ankle kinematic parameters when DF-FES was turned on (vs. off) at both the first (after one month) and final (after five months) gait analysis, including the initial contact angle (*p <* 0.01) and minimal and maximal dorsiflexion during the mid and terminal swing (*p <* 0.01) (Table 3).

Analyzing the kinematic effect of the device at the first gait analysis (N = 22), this effect varied among the patients. A total of 11 of 22 patients (50%) showed an orthotic effect (OE+), with plantarflexion (<0°) prevented during the mid and/or terminal swing, while the other 11 patients showed no orthotic effect (OE–), with no correction of the foot drop (see Supplementary Table S1).

Six (85%) of the seven patients who dropped out after the initial gait analysis were OE–. At the final gait analysis, 12 (80%) of 15 patients demonstrated an orthotic effect, and 3 (20%) failed to achieve OE, with no difference in compliance (*p* = 0.6).

#### 3.4.2. Predictors of OE+ and OE− at First Gait Analysis

Supplementary Table S2 shows different baseline variables for participants who did and did not gain an orthotic effect at the first gait analysis. There were no significant differences between the 11 patients who showed an orthotic effect and the 11 who did not, in terms of demographic, device use, clinical, kinematic, or biomechanical variables, including passive ankle ROM (knee extension/flexion). The only significant difference found between the subgroups was ankle dorsiflexion at mid-stance (MS-DF), which was significantly correlated with the presence of OE at gait analysis (r = 0.56, *p <* 0.01). Specifically, children in the OE+ group had greater ankle dorsiflexion at mid-stance compared to the OE– group (median 20.37° (15.87, 22.05) vs. 11.75° (10.1, 16.66), respectively, *p <* 0.01).

#### 3.4.3. Kinematic Parameters

No statistical change was noted in the study group with respect to the first and final ankle kinematic parameters (off vs. off and on vs. on) (Table 3).

#### 3.4.4. Spatiotemporal Parameters

Comparing the spatiotemporal parameters of the full study group (N = 15) between the first and final gait analysis, there was no evidence for the normalization of gait deviations, with no significant change in the proportions of the gait cycle comprising the stance phase nor a decrease in the double/single support ratio. In contrary to our primary hypothesis, there were indications of slower walking, including an increase in step time (0.49 s (0.43, 0.54) vs. 0.53 s (0.5, 0.59) for the first and final gait analysis, respectively, with DF-FES off, *p <* 0.05), and a trend toward a decrease in cadence (steps/min; *p* = 0.06) with no change in step length. Still, in direct measures of walking speed, the decrease was not statistically significant (Table 3).

The indications of slower walking grew stronger when comparing the spatiotemporal parameters between the first and final gait analysis only for the twelve patients who gained OE (at the final analysis). We found a significant increase in the stance time (sec), a decrease in cadence (steps/min), and an increase in the double/single support ratio (supported gait). Specifically, the stance times were 0.52 (0.46, 0.56) vs. 0.56 (0.53, 0.64) sec; the cadence was 128.4 (123.2, 146.1) vs. 120 (108.6, 127.4) steps/min; and the double/single support time ratio was 0.29 (0.25, 0.31) vs. 0.32 (0.29, 0.34), all for the first and final gait analyses, respectively (DF-FES off; *p <* 0.05).

#### 3.4.5. Biomechanical Parameters

Neither the ankle MAS score, ankle passive ROM, nor muscle strength of the study group changed statistically between the baseline and final assessment. However, some patients did show an improvement in these parameters. For example, seven (46%) and nine (60%) improved their ankle ROM with the knee flexed and knee extended, respectively (see Supplementary Table S3).

## 4. Discussion

This prospective open-label study assessed the effects of five months of DF-FES use on aspects of daily motor functioning, such as stability and postural control, which are impaired in high-functioning (GMFCS I/II) children with hemiplegic CP. The findings demonstrated a significant change over time in the Community Balance and Mobility Scale, with a median difference of 6.5 points (95% CI (2.79, 10), *p <* 0.01) after five months of device use, where a change of five points is considered clinically meaningful [16]. The CB&M was developed to evaluate the balance and mobility of patients who may be ambulatory yet still have balance and mobility deficits. Wright and Bos [28] showed that even children with typical development normally do not reach the maximal score, so this test may be used in high-functioning children with hemiplegia who reach a ceiling effect on other objective measures [18]. Similar to our results, Pool et al. [16] showed an improvement in this scale of 8.3 units (95% CI [3.2, 13.4]) compared to a control group after eight weeks of FES use in children with hemiplegic CP. It should be noted that significant improvement was noticed in the present study already after four weeks of device use. After the first month, improvement continued, although the difference between month one and month five was not significant. In addition, falling frequency questionnaires filled out by parents revealed a trend toward improvement in stability, although those results were not statistically significant (*p* = 0.06).

Pool et al. [16,18] found improvement in ankle biomechanical parameters, including spasticity, range of motion, and muscle strength, under continuous DF-FES use. These biomechanical improvements may account for better postural stability. In addition, Pool [18] hypothesized that the repetitive motion of the ankle leads to improved reciprocal inhibition, reduced muscle co-activation, and better coordinated muscle activation. Our study found no statistically significant changes in either ankle biomechanical parameters or kinematic parameters over time. Still, the absence of statistical change may have other causes, such as the limited sensitivity of methods such as manual muscle testing for detecting minor changes in muscle power [35] or reduced power due to our small study sample. Taking a close look at the particular biomechanical parameters of the patients at baseline and final assessments (Supplementary Table S3), we can see that many patients did show improvement in some of their biomechanical parameters. Larger studies are needed to define more precisely which patients may or may not improve their biomechanical parameters.

As noted in the introduction, studies examining the therapeutic effects of DF-FES (namely, any improvement after the continuous use of DF-FES that continues without the device) have been inconclusive [13]. Bailes [17] found evidence for a therapeutic effect in some but not all parameters of the SWOC (Standardized Walking Obstacle Course test) after four months of DF-FES use. Our findings, like those of Pool [18], showed improvement over time in the CB&M test with DF-FES turned off, implying at least an immediate therapeutic effect. Khamis [13] argued that the retention effect is probably temporary and dependent on the continuous use of FES, but Pool [18] showed that the effect lasts for at least six weeks post-treatment. More studies are needed to evaluate the persistence of this carry-over effect.

We used the TUDS test as another method to assess postural control in the population of children with CP. This test requires a certain strength in the lower extremities and trunk, ROM in the lower extremities, coordination during fast reciprocal movements, and postural control. The present findings show only a trend toward minor improvement in the TUDS. A lack of a significant change in biomechanical parameters such as ankle ROM may explain the lack of significant improvement in the TUDS test.

Our findings with respect to the 6MWT call for close scrutiny. Bailes [19] reported a mean increase of 52 m in the 6MWT after four months on DF-FES, which may imply increased physical endurance. Contrary to our primary hypothesis, in our study, the 6MWT revealed the opposite trend, with a statistically decreased walking distance of 30 m (DF-FES off) over time. This finding seems to reflect a change toward slower walking speeds. Previous work suggests a minimum detectable change of 61.9 m in the 6MWT for GMFCS Level I, [33], which is not met by our study. However, the changes in spatiotemporal parameters point to a real trend. While there was no improvement in spatiotemporal deviations towards the norm, there were several indications of slower walking, especially in the subgroup that gained an orthotic effect. In this subgroup, there was a decrease in cadence (steps/min) with no change in step length, an increase in step time, an increase in stance time, and an increase in the double/single support ratio. Taken together, the data point to slower walking, even though in a direct measure of walking speed across a 14 m walkway, this trend was not statistically significant. Walking for a longer distance, as in the 6MWT, may be needed to notice a significant change.

Indeed, the literature is characterized by conflicting results regarding the effect of FES on walking speed in children with CP, with some results showing an increase [17,23], others a decrease [25], and others no change [24]. The implications and causes of changes in walking speed, and, in particular, reduced walking speed, are also unclear. One possibility is that slower walking may reflect a negative effect of DF-FES. Bailes et al. [19] reported a deterioration over time in kinematic parameters (e.g., a decrease in peak swing dorsiflexion) during DF-FES use. They hypothesized that this deterioration may derive from a lack of voluntary muscle effort and weakness among patients who rely on the device [10,19]. Another explanation for slower walking could be a natural decline in functional capacity over time in the CP population. However, our study did not show a decrease in kinematic parameters over time or muscle weakness. In addition, the reduction in walking distance was noted very early, already after one month, which implies that the change should be attributed to the device rather than natural decline.

On the other hand, slower walking also has some advantages. Van der Linden et al. [25] suggested that since many children with CP have difficulty controlling their forward progression, a decrease in speed may reflect a more controlled gait pattern. Slower walking causes an increase in the double support phase, both in time and as a percentage of the gait cycle. During double support, stability is increased since patients have more control over their center of mass movement [39]. Children with hemiplegia have a higher baseline double/single support ratio than typically developing children [6]. It seems that DF-FES does not repair this deviation, but, by causing slower walking, it increases stability.

Patients did not experience the device as annoying or uncomfortable, so a slower gait is probably not attributable to adverse effects of the electrical stimulation. In addition, the decrease in distance appeared even when DF-FES was turned off. Damiano [10] showed improvement in max swing DF over time only at self-selected speeds, but not at the patient’s fastest walking speed. It may be that inherent features of the device limit a full dorsiflexion effect during fast walking, leading patients to walk slower in order to obtain the full effect of the device. This may reflect a limitation of the device, as it lacks a closed loop control system that would allow for adaptation to different walking speeds [40]. On the other hand, it may encourage patients to adopt a slower and more controlled gait pattern, even in the DF-FES off state. Larger and directed studies are needed to test the orthotic effect at different walking speeds.

There are currently no clinical tools able to identify appropriate candidates for DF-FES in children with hemiplegic CP. In this study, only 58% of eligible patients completed the study protocol. Patients who dropped out had a significantly higher prevalence of previous botulinum toxin injections and a non-significant trend toward a higher prevalence of AFO use. These findings may imply a worse baseline condition in those patients. It is noteworthy that the high drop-out rate was related mostly to the inefficacy of the device in producing an orthotic effect. Six (85%) of seven patients who dropped out after the first gait analysis did not achieve swing ankle dorsiflexion ≥0° at either the mid or terminal swing. Our findings emphasize that a precondition for gaining a functional benefit from the continuous use of DF-FES is an achievement of an orthotic effect; otherwise, compliance will be poor. Khamis [13] noted that while no absolute criteria were found to predict the suitability of FES devices, a prerequisite is the ability to maintain adequate passive dorsiflexion; however, they left open the precise meaning of “adequate.” Previous studies have used different patient inclusion criteria [5]—e.g., passive ankle ROM with knee extension above 0° [19] or 5° [18].

With the aim of identifying predictors for the achievement of an orthotic effect, we compared the demographic, device use, and clinical parameters of those who did (OE+) or did not (OE–) achieve such an effect at the first gait analysis. We hypothesized that those with a better ankle ROM would gain an orthotic effect (OE+). Although passive ankle ROM angles were not significant predictors for the presence or absence of OE, we found that a larger dorsiflexion angle during the mid-stance is a significant predictor for OE+. We looked specifically at this parameter as another indication for ankle ROM during the weight-bearing state [41]. This parameter may reflect the functional ankle ROM better than a passive ankle ROM test. Thus, adequate ankle ROM, as a prerequisite for using DF-FES, should not be measured passively but rather during gait or through other weight-bearing measures [42,43]. Among the 15 patients who completed the study, 3 failed to achieve OE, although no difference in compliance was noted. Two of those who did not gain OE had a limited mid-stance angle. Larger studies are needed to define other predictors for orthotic effect achievement and appropriate candidates for this intervention.

This study has limitations. Some of our borderline statistical results (e.g., falling frequency) may reflect limited power due to the small size of the study cohort. Methodologically, this was an open-label study with no control group. This limitation may raise the possibility that the improvement observed in balance scores relates to the effects of time and practice and not to the DF-FES intervention. However, the early change in balance scores, together with walking distance, implies the true influence of the device.

The timing of the first gait analysis is another potential limitation, since it was conducted after one month of device use and not at the true baseline. This decision was made a priori (at study protocol), based on previous work [12,19,24] suggesting that a period of adjustment to the device is necessary in order to assess the true orthotic effect at gait analysis. Due to budget limitations we chose to conduct two gait analyses, after one month and after five months of use, on the assumption—supported by previous studies [10,19]—that baseline kinematic and spatiotemporal parameters would not change significantly during the first month of intervention. If this assumption is incorrect, significant changes in kinematic parameters could have been missed.

It should also be noted that this study focused on changes in kinematic parameters on the affected side. Changes on the unaffected side should be tested further.

## 5. Conclusions

The continuous use of DF–FES produces an early functional benefit and immediate therapeutic effect with better stability and postural control. A precondition for gaining a functional benefit from the continuous use of DF-FES is the achievement of an orthotic effect; otherwise, compliance will be poor. Limited mid-stance dorsiflexion can serve as a predictor for a failure to achieve an orthotic effect. Results regarding the walking speed are still inconclusive; however, we found indications that, over time, walking becomes slower, especially in those who gain an orthotic effect. While this may imply a negative outcome, slower walking is more supported and controlled, which may contribute to improved stability.

## Figures and Tables

**Table 1:** Clinical and demographic parameters of the study group and patients who withdrew.

Parameters	Study Group (N = 15)	Withdrawn Patients (N = 11)
Age (y)	8.2 (7, 10.5)	8 (6.8, 8.8)
M:F	10:5	5:6
Term (≥37 w)	7 (47%)	6 (55%)
GMFCS I:II	14:1	8:3
Current AFO use	7 (46%)	7 (63%)
Botulinum toxin—LL		
No	12 (80%)	3 (27%)
Yes	3 (20%)	8 (73%) *
Surgery to LL (n)		
No	14 (93%)	9 (82%)
Yes	1(7%)	2 (18%)
MAS		
1	2 (13%)	3 (27%)
1+	4 (27%)	2 (18%)
2	9 (60%)	6 (55%)
Passive ankle ROM		
Knee flexion (degrees)	10° (5, 15)	10° (7.5, 15)
Knee extension (degrees)	5° (2, 9)	5° (1, 10)

* *p <* 0.05 (Fisher’s exact test). M = Male; F = Female; AFO = ankle foot orthoses; LL = lower limb; MAS = Modified Ashworth Scale; ROM = range of motion.

**Table 2:** Motor function scores—median difference.

Tests		Median Difference (95% CI) *		
		Baseline to 1 Month	1 Month to 5 Months	Baseline to 5 Months
**CB&M** **(score)** **(N = 14)**	**DF-FES off**	3 ^$^	3	6 ^$^
		(1, 7.2)	(−3.2, 4.1)	(1.89, 8.1)
	**DF-FES on**	4.5 ^$^	1	6.5 ^$^
		(0.89, 8.3)	(−4, 7.1)	(2.79, 10)
**6 MWT** **(meter)** **(N = 15)**	**DF-FES off**	−17.5	−17.5	−30 #
		(−67.08, 15)	(−73.12, 16.04)	(−83.67,−2.6)
	**DF-FES on**	−30 #	−12.5	−35 #
		(−55, −4.47)	(−45.5, 11.04)	(−99.67, −3.97)
**TUDS** **(sec)** **(N = 15)**	**DF-FES off**	−0.19	−0.19	−0.83
		(−2.34, 0.4)	(−0.76, 0.7)	(−2.28, 0.42)
	**DF-FES on**	−0.7	−0.68	−0.41
		(−1.71, 1.19)	(−1.71, 0.4)	(−2.7, 0.28)

* Main comparisons are between baseline scores (DF-FES off) and one- or five-month scores (DF-FES off and on). CB&M = Community Balance and Mobility Scale; 6MWT = Six-Minute Walk Test; TUDS = Timed Up and Down Stairs Test. ^$^ *p* < 0.01; # *p* < 0.05 (Wilcoxon test).

**Table 3:** Ankle kinematic and spatiotemporal parameters with DF-FES off and on at the first and final gait analyses.

	DF-FES off (N = 15)		DF-FES on (N = 15)	
FES	First	Final	First	Final
Maximal dorsiflexion—mid swing (degrees)	−4.57°	−3.3°	3.13° *	3.2° #
	(−9.1, 4.63)	(−10.05, 4.06)	(−5.97, 6.01)	(−4.04, 6.64)
Maximal dorsiflexion—terminal swing (degrees)	−3.52°	−3.86°	3.97° *	3.36° #
	(−7.53, 2.05)	(−7.77, 1.79)	(−0.39, 6.49)	(1.64, 7.62)
Minimal dorsiflexion—mid swing (degrees)	−11.68°	−6.72°	−0.97° *	−0.58° #
	(−15.07, 0.74)	(−16.5, −0.99)	(−11.62, 1.63)	(−9.95, 3.28)
Minimal dorsiflexion—terminal swing (degrees)	−11.46°	−11.24°	−1.88° *	−1.6° #
	(−14.5, −4.74)	(−15.59, −7.68)	(−6.28, 1.46)	(−6.82, 0.94)
Initial contact (degrees)	−7.08°	−7.2°	−0.49° *	0.81° #
	(−9.1, −1.02)	(−11.78, −3.79)	(−4.32, 2.99)	(−2.33, 2.7)
Peak swing dorsiflexion (degrees)	−0.82°	−2.18°	4.51° *	4.27° #
	(−6.59, 4.89)	(−4.3, 4.22)	(0.08, 7.18)	(1.94, 8.03)
Stance time (sec)	0.53	0.56	0.52	0.56
	(0.46, 0.57)	(0.53, 0.63)	(0.49, 0.61)	(0.51, 0.59)
Stance—%gait cycle	56.88	57.42	56.29	56.54
	(55.65, 57.59)	(56.59, 57.72)	(54.76, 56.96)	(55.61, 57.23)
Walking speed (meter/sec)	1.09	1.04	1.1	1.07
	(0.955, 1.260)	(0.97, 1.13)	(0.95, 1.2)	(0.96, 1.17)
Cadence (steps/min)	127.9	122.4	126.32	118.44
	(120.12, 147.16)	(111.42, 127.54)	(115.91, 134.91)	(112.69, 131.87)
Double/single support time ratio	0.42	0.47	0.42	0.4
	(0.35 to 0.46)	(0.4 to 0.53)	(0.36 to 0.45)	(0.36 to 0.44)
Step length (cm)	0.49	0.51	0.51	0.5
	(0.42, 0.52)	(0.46, 0.53)	(0.48, 0.57)	(0.46, 0.53)
Step time (sec)	0.49	0.53 $	0.52	0.54
	(0.43, 0.54)	(0.5, 0.59)	(0.48, 0.54)	(0.48, 0.5)

Data are presented as median degrees (interquartile range (IQR)); * First gait analysis: FES off vs. FES on, *p <* 0.01; # Final gait analysis: FES off vs. FES on, *p <* 0.01. $ Final vs. first gait analysis, *p <* 0.05 (Wilcoxon test).

## Data Availability

The authors declare that the data supporting the study findings are available within the paper and its additional file. The remaining data are available from the corresponding author upon reasonable request.

## Supplementary Materials

The following supporting information can be downloaded at https://www.mdpi.com/article/10.3390/children10030531/s1, Table S1: Kinematic parameters of patients showing (OE+) and not showing OE (OE–) at first gait analysis; Table S2: Clinical and demographic parameters of patients showing (OE+) and not showing OE (OE–) at first gait analysis; Table S3: Biomechanical parameters at baseline and final assessments of the study group.
