# Supplementary material for: Functional Benefit and Orthotic Effect of Dorsiflexion-FES in Children with Hemiplegic Cerebral Palsy
Source: Children (Basel). 2023 Mar 9;10(3):531. doi: 10.3390/children10030531 (PMC10047387; doi:10.3390/children10030531)
Supplement: Supplementary file 1 [file children-10-00531-s001.zip › Table S1 (Supplementary) ankle Kinematic parameters of subgroups with or without OE _minor revesion.pdf]

Supplementary Table S1. Kinematic parameters of patients showing (OE+) and not showing OE (OE–) at first gait analysis

	OE+ (N=11)		OE- (N=11)	
**FES**	**Off**	**On**	**Off**	**On**
**Maximal dorsiflexion – mid swing (degrees)**	0.06° (-8.74, 4.42)	3.85° * (0.57,5.64)	-7.39° (-10.13, -3.7)	-5.99° (-9.27, -2.97)
**Maximal dorsiflexion – terminal swing (degrees)**	-2.39° (-6.86, 3.47)	4.55° * (3.76, 6.54)	-4.90° (-7.96, -2.19)	-4.38° (-6.09, -1.17)
**Minimal dorsiflexion –mid swing (degrees)**	-11.21° (-18.99, 1.07)	0.15° * (-10.91, 1.6)	-11.68° (-14.68, -8.4)	-11.5° (-13.67, -5.84)
**Minimal dorsiflexion – terminal swing (degrees)**	-9.8° (-14.93, -3.9)	0.36° * (-2.72, 2.10)	-12.1° (-13.8, -9.34)	-8.06° (-11.6,-5.16)
**Initial contact (degrees)**	-3.58° (-7.63, -0.64)	2.01° * (0.35, 3.08)	-8.09° (-9.64, -4.24)	-5.73° (-7.12, -3.86)

Data are presented as median degrees (interquartile range [IQR]); *FES off vs on, $p<0.01$, ** FES off vs on, $p<0.05$.

OE=orthotic effect
