# Supplementary material for: Functional Benefit and Orthotic Effect of Dorsiflexion-FES in Children with Hemiplegic Cerebral Palsy
Source: Children (Basel). 2023 Mar 9;10(3):531. doi: 10.3390/children10030531 (PMC10047387; doi:10.3390/children10030531)
Supplement: Supplementary file 1 [file children-10-00531-s001.zip › Table S2 minor revesion_(Supplementary )2 Comparison of clinical and demographic parameters of patients with OE and without OE.pdf]

Supplementary Table S2. Clinical and demographic parameters of patients showing (OE+) and not showing OE (OE–) at first gait analysis

	OE+(N=11)	OE– (N=11)
Age (y)	8.30 (7, 10.5)	8.00 (7.5, 9.80)
Male:Female	8:3	5:6
Birth (≥37w)	6 (55%)	5 (45%)
Etiology		
Arterial infarct	4 (36.5%)	3 (27.5%)
Venous infarct	4 (36.5%)	5 (45.5%)
White matter injury	2 (18%)	1 (9%)
Unknown	1 (9%)	2 (18%)
GMFCS I/II	11:0	10:1
Current AFO use	5 (45.5%)	6 (54.5%)
Botulinum toxin -LL		
-No	8 (73%)	5 (45%)
-Yes	3 (27%)	6 (55%)
Surgery to LL(n)		
-No	10 (91%)	9 (82%)
-Yes	1 (9%)	2 (18%)
Device use Stimulation		
intensity	3 (3, 4)	3.5 (2.75, 5)
Hours/day	5.51 (4.84, 7.06)	5.73 (5.17,6.03)
Stims/day	3432 (2072, 3850)	3067 (2715, 3589)
MAS		
1	1 (9%)	4(36%)
1+	2 (18%)	3(28%)
2	8 (73%)	4(36%)
Muscle strength		
2	2 (18%)	3 (28%)
3	2 (18%)	4 (36%)
4	4 (36%)	3 (28%)
5	3 (28%)	1 (9%)
Muscle selectivity		
0	2 (18%)	1 (9%)
1	4 (36%)	8 (72%)
2	5 (45.5%)	2 (18%)
Passive ankle ROM		
Knee flexion (deg)	10° (5.5, 15)	10° (5, 10)
Knee extension (deg)	5° (2, 8.75)	2° (0, 7.25)
Kinematic parameters#		
Heel strike	2° (18%)	1° (9%)
Max Swing DF (deg)	-5.35° (-8.06,-4.31)	-4.8° (-7.8,-3.43)
Initial contact (deg)	-6.5° (-9.38,-4.56)	-3.18° (-8.05,-1.15)
Midstance Max Dorsiflexion (deg)	20.37° (15.87, 2.05)	11.75° (10.1, 16.66)*

Data are presented as median (interquartile range [IQR]) or number (%). * OE+ vs OE–, p<0.01. (Mann-Whitney test). # kinematic parameters (degrees) of patients while DF-FES is turned off. OE=orthotic effect; GMFCS=Gross Motor Function Classification System; AFO=ankle-foot orthosis; Deg= degrees; DF=Dorsiflexion; LL=lower limb; MAS=Modified Ashworth Scale; ROM=range of motion; Max=maximal
