# Supplementary material for: Functional Benefit and Orthotic Effect of Dorsiflexion-FES in Children with Hemiplegic Cerebral Palsy
Source: Children (Basel). 2023 Mar 9;10(3):531. doi: 10.3390/children10030531 (PMC10047387; doi:10.3390/children10030531)
Supplement: Supplementary file 1 [file children-10-00531-s001.zip › Table S3 (supplementary) comparison of biomechanical parameters.pdf]

Supplementary Table S3: Biomechanical parameters at baseline and final assessments of study group.

Patient	Ankle ROM (knee flexed) (degrees)		Ankle ROM (knee extended) (degrees)		MAS score		Muscle strength		Muscle selectivity	
	**Baseline**	**Final**	**Baseline**	**Final**	**Baseline**	**Final**	**Baseline**	**Final**	**Baseline**	**Final**
**1**	5°	10°	5°	10°	1+	1+	3	4	0	0
**2**	5°	5°	2°	5°	2	2	5	5	2	2
**3**	15°	2°	5°	2°	1+	1+	2-	2-	1	1
**4**	10°	15°	0°	5°	2	1+	2-	2	1	1
**5**	25°	25°	20°	20°	1	1	3-	2	1	1
**6**	15°	20°	8°	10°	1+	1+	3	2+	1	1
**7**	10°	15°	2°	5°	2	1+	3+	3+	2	2
**8**	15°	10°	5°	10°	2	2	4	4	2	2
**9**	20°	20°	15°	20°	1	2	4-	4	1	1
**10**	2°	20°	0°	10°	2	1+	4	4	2	2
**11**	5°	10°	2°	4°	1+	1	5	5	2	2
**12**	2°	5°	0°	0°	2	2	2-	2-	1	1
**13**	7°	5°	5°	5°	2	2	2	2	2	2
**14**	15°	15°	15°	15°	2	1	4	4	0	1
**15**	10°	10°	10°	5°	2	1+	3	3	2	2

ROM=range of motion; MAS=Modified Ashworth Scale.
